# Trimethylation of H3K27 during human cerebellar development in relation to medulloblastoma

**DOI:** 10.18632/oncotarget.20741

**Published:** 2017-09-08

**Authors:** Shahryar E. Mir, Michiel Smits, Dennis Biesmans, Machteld Julsing, Marianna Bugiani, Eleonora Aronica, Gertjan J.L. Kaspers, Jacqueline Cloos, Thomas Würdinger, Esther Hulleman

**Affiliations:** ^1^ Department of Pediatric Oncology/Hematology, VU University Medical Center, Amsterdam, The Netherlands; ^2^ Neuro-oncology Research Group, Departments of Neurosurgery and Pediatric Oncology/Hematology, VU University Medical Center, Amsterdam, The Netherlands; ^3^ Department of Pathology, VU University Medical Center, Amsterdam, The Netherlands; ^4^ Department of (Neuro) Pathology, Academic Medical Center and Swammerdam Institute for Life Sciences, Center for Neuroscience, University of Amsterdam, Amsterdam, The Netherlands; ^5^ Department of Hematology, VU University Medical Center, Amsterdam, The Netherlands; ^6^ Department of Neurology, Massachusetts General Hospital and Harvard Medical School, Boston, MA, USA

**Keywords:** medulloblastoma, cerebellum, brain development, histone 3 trimethylation, immunohistochemistry

## Abstract

Medulloblastoma (MB), the most common malignant childhood brain tumor, encompasses a collection of four clinically and molecularly distinct tumor subgroups, i.e. WNT, SHH, Group 3 and Group 4. These tumors are believed to originate from precursor cells during cerebellar development. Although the exact etiology of these brain tumors is not yet known, histone modifications are increasingly recognized as key events during cerebellum development and MB tumorigenesis. Recent studies show that key components involved in post-translational modifications of histone H3 lysine 27 (H3K27) are commonly deregulated in MB. In this descriptive study, we have investigated the trimethylation status of H3K27, as well as the expression of the H3K27 methylase EZH2 and demethylases KDM6A and KDM6B, during human cerebellum development in relation to MB. H3K27 Trimethylation status differed between the MB subgroups. Moreover, trimethylation of H3K27 and expression of its modifiers EZH2, KDM6A and KDM6B were detected in a spatio-temporal manner during development of the human cerebellum, with consistent high occurrence in the four proliferative zones, which are believed to harbor the precursor cells of the different MB subgroups. Our results suggest that H3K27 trimethylation in MB is deregulated by EZH2, KDM6A and KDM6B. Moreover, we provide evidence that during development of the human cerebellum H3K27me3 and its regulators are expressed in a spatio-temporal manner.

## INTRODUCTION

During development, cerebellar neurons are generated from two anatomically and molecularly distinct progenitor zones: the cerebellar ventricular zone (VZ) and the rhombic lip (RL) [1–3]. The VZ gives rise to all the GABAergic neurons, including Purkinje cells and inhibitory interneurons. The RL gives rise to all glutamatergic neurons, including granule neuron precursor cells (GNPs), unipolar brush cells and deep nuclei neurons. Exiting the RL, GNPs migrate rostrally across the pial surface of the cerebellum and form a secondary germinal zone, the external germinal layer (EGL). This cell layer proliferates extensively until the first months postnatal, producing granule cells which reside in the internal granule layer (IGL). These proliferative zones are present during cerebellar development at a specific spatio-temporal manner [2, 3]. Medulloblastomas (MB) are highly invasive primitive neuroectodermal tumors, which are believed to originate from aberrantly dividing precursor cells present during cerebellar development [4]. MB are considered to encompass a collection of four clinically and molecularly distinct tumor subgroups [5–7]. For two of these subgroups the cell of origin has been identified. The first subgroup has aberrant activation of the Sonic Hedgehog (SHH) pathway and originates from GNPs of the developing cerebellum [8, 9]. The second subgroup has activating mutations in the WNT pathway effector beta-catenin 1 (CTNNB1) and arises outside the cerebellum from cells of the lower RL located at the dorsal brainstem during early hindbrain development [10]. For the other MB subgroups, i.e. Group 3 and Group 4, the pathological processes that drive tumor formation remain elusive, although these tumors show GABAergic (Group 3) and glutamatergic features (Group 4) [11–13].

Epigenetic gene regulation is essential for neural differentiation and crucial during human cerebellum development [14–16]. Histone modifications, such as histone acetylation, are associated with active gene transcription, whereas others such as the trimethylation of histone H3 lysine 27 (H3K27me3) are an indicator of condensed and inactive chromatin [17]. Important regulators of histone modification are the polycomb group protein complexes. The *Enhancer of zeste homolog 2* (EZH2) is an essential member of these complexes, as it has methyltransferase activity. EZH2 specifically trimethylates H3K27, leading to target gene silencing [18]. Counterparts of these repressive complexes are the histone demethylases KDM6A and KDM6B, which can specifically remove methyl marks of H3K27, and are able to activate silenced genes [19–21].

Deregulation of H3K27 trimethylation has recently been identified in all subgroups of MB [22–25]. Genome-wide mutation analyses in MBs have identified inactivating mutations in *KDM6A* and *KDM6B*, the two demethylases of H3K27, in respectively 8% and 0.3% of sequenced MB [22, 23]. Moreover, *KDM6A* (Xp11.3) and *KDM6B* (17p13.1) are also affected at a copy number level as deletions have been identified in almost 30% of MB cases [23]. Overexpression of EZH2 has been reported in several types of cancers, [26–29], including MB where *EZH2* (7q36.1) is gained and overexpressed in almost 14% of MB cases [23]. Moreover, functional studies have provided compelling evidence that targeting EZH2 in MB activates silenced tumor suppressor genes and reduces stem cell properties in MB cell lines and transforming capacity in neural stem cells [30, 31]. Although deregulation of H3K27me3 occurs in all MB subgroups, the strongest effects are observed in Group 3 and Group 4 MB [23–25]. Importantly, in these subgroups increased trimethylation of H3K27 leads to poor outcome [23]. While H3K27 methylation status seems to play an important role in MB, its role during cerebellum development has not been evaluated previously. Since MB are believed to arise from aberrantly dividing precursor cells present during cerebellar development, in this descriptive study we have investigated the expression of EZH2, KDM6A, KDM6B and the H3K27me3 at various developmental stages of the human cerebellum.

## RESULTS

### H3K27me3 and its regulators are expressed differentially in MB subgroups

**Figure 1:**
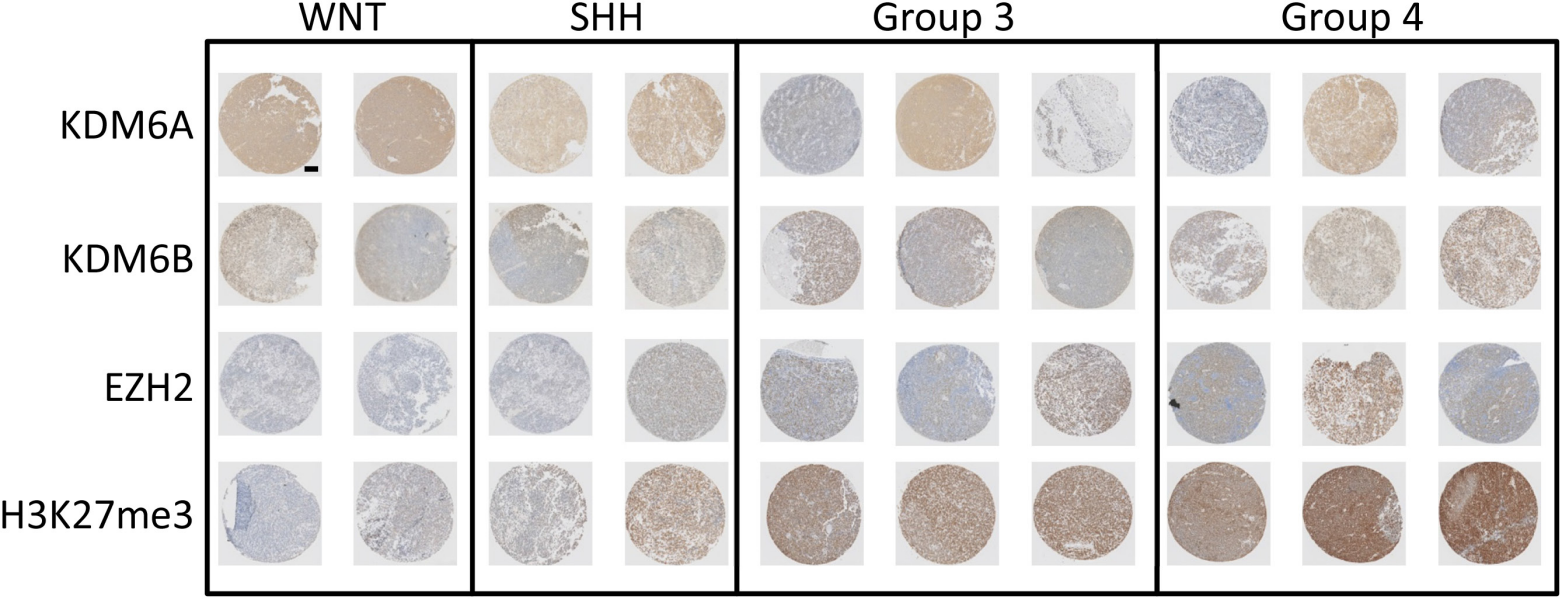
Expression of H3K27 and its modifiers in medulloblastoma Representative selection of immunohistochemical staining for KDM6A, KDM6B, EZH2 and H3K27me3 in MB samples of different subgroups.

We evaluated the presence of the chromatin H3K27me3 mark and the expression of the methylase EZH2 and demethylases KDM6A and KDM6B in a selection of MB samples with known subgroup status. A subgroup specific expression pattern was observed, with increased trimethylation for H3K27 and increased EZH2 expression in group 3 and group 4 MB samples (Figure 1). The increased H3K27me3 in these subgroups also correlated with low protein expression of KDM6B. The expression of KDM6A was relatively high in most samples and did not correlate with any subgroup. Intermediate expression for EZH2 and H3K27me3 was found for SHH medulloblastoma samples, with both low and high expression of KDM6A and KDM6B. The WNT group samples had the lowest expression of EZH2 and H3K27me3, combined with relatively higher KDM6A and KDM6B protein levels (Figure 1). These results are in line with previous studies showing aberrant trimethylation of H3K27 in a subgroup-specific manner in MB [23, 24].

### The expression of H3K27me3 and its regulators in developing human cerebellum

MB is considered to arise through transformation of early developmental progenitor cells [4]. To identify potential precursors for H3K27 deregulated MB, we evaluated the presence of H3K27 markers during human cerebellum development.

**Figure 2:**
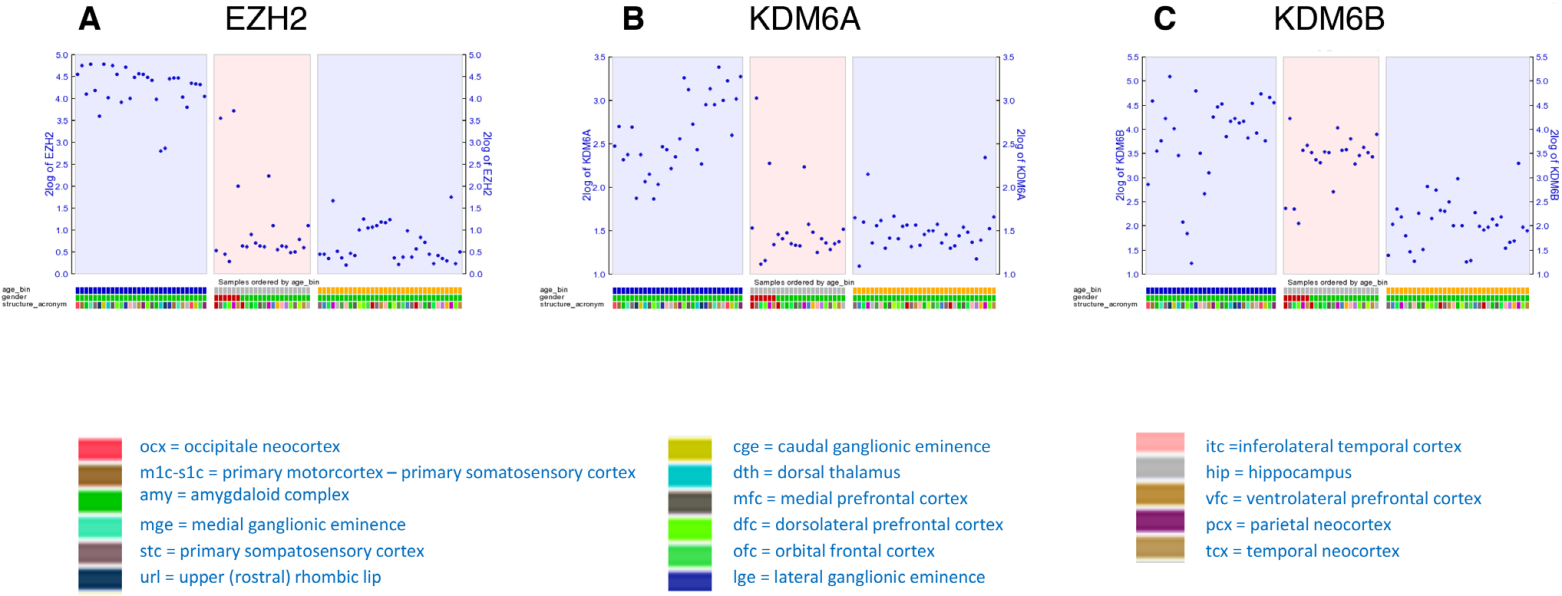
Dot plot showing transcript levels of EZH2 **(A)**, KDM6A **(B)** and KDM6B **(C)** during human development as reported in the Brainspan database. Depicted are stage 2A (corresponding with 9 weeks GSA; early prenatal – blue), stage 5 (corresponding to 28 and 33 weeks GSA; late prenatal – gray), and stage 6 (corresponding to 40 weeks GSA; birth-early infancy – yellow).

First, we analyzed transcriptional data previously published as part of the BrainSpan Atlas of the Developing Human Brain (http://brainspan.org/) [32]. The gene expression data for *EZH2*, *KDM6A* and *KDM6B* were analyzed for the periods spanning from embryonic (9 weeks GSA) until birth/early infancy (40 weeks). Throughout the prenatal period EZH2 displayed high mRNA expression in the developing human brain. This expression then dropped significantly after birth, with consistent low expression onwards (Figure 2A). KDM6A was also highly expressed prenatally, although expression varied between different brain regions and samples (Figure 2B). KDM6B expression was less consistent during prenatal development, with a trend towards lower expression during postnatal periods (Figure 2C).

To further evaluate the trimethylation status of H3K27 in human cerebellum development, we established a cohort of human cerebellum samples during different time points of development, ranging from gestational age (GSA) 9 weeks till 40 weeks. At least two samples per gestational age were investigated, with no difference in expression between samples.

**Figure 3:**
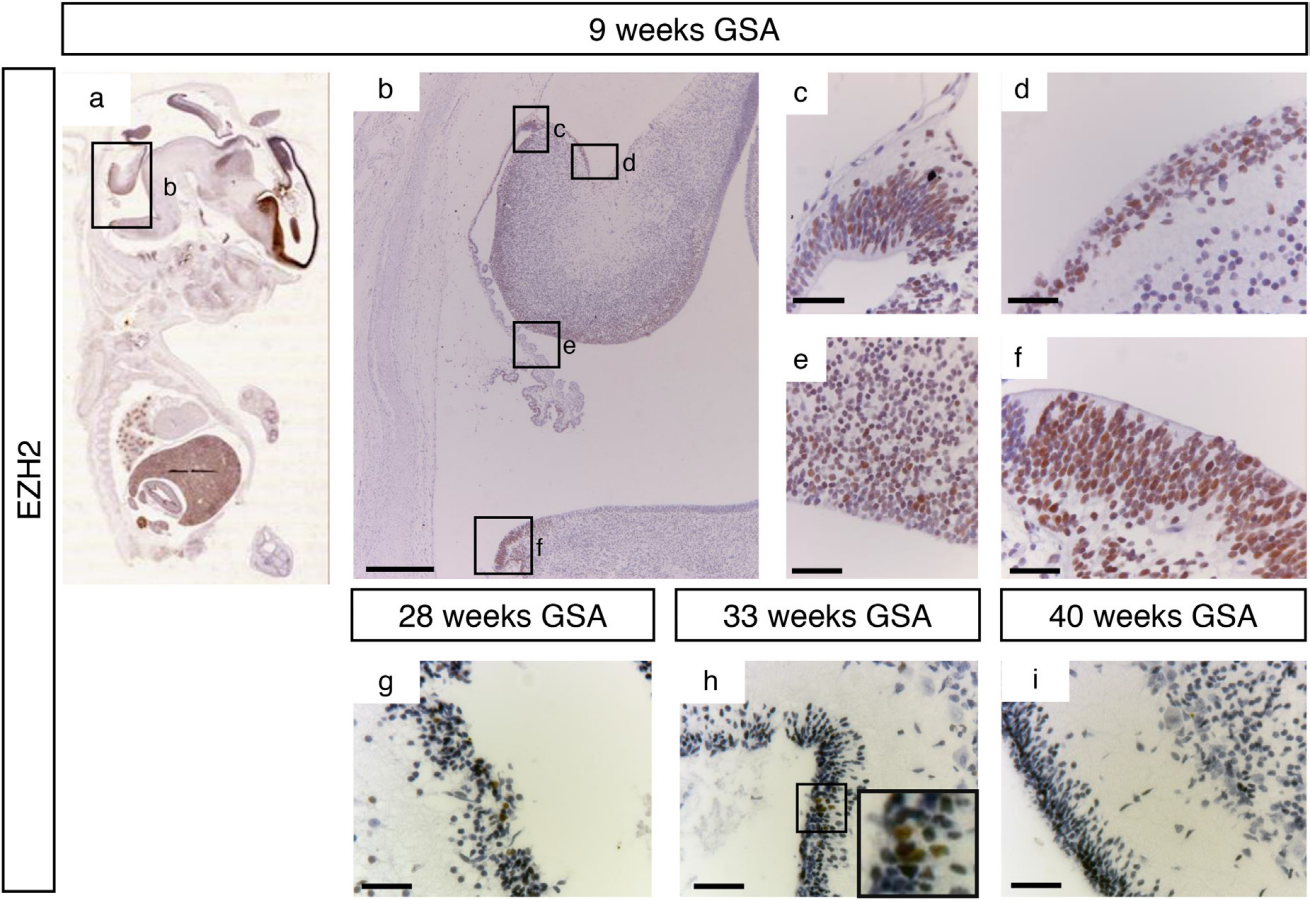
EZH2 expression during human cerebellum development **(a)** Expression of EZH2 at 9 weeks GSA. **(b-f)** Higher magnifications (400x) of boxed areas showing high EZH2 expression in the proliferative zones of the developing cerebellum; upper RL (c), EGL (d), VZ (e), and lower RL (f). Expression of EZH2 is only detectable in the EGL layer between 28 weeks gestation **(g)** and 33 weeks gestation (h). Higher magnification of boxed area in **(h)** shows the EZH2 positive cells in the EGL layer. After 33 weeks no EZH2 expression can be detected in the cerebellum **(i)**. Scale bars 1000 μm (b), 25 μm (c-i), 5 μm (inset in h).

At 9 weeks of gestation all important proliferative zones could be visualized (the VZ, upper- and lower RL and the EGL). Staining with an antibody directed against EZH2 showed a strong nuclear signal in all these different zones (Figure 3a–3f). However, at later time points EZH2 expression was markedly reduced and restricted. Between 28 weeks and 33 weeks gestation EZH2 was only detectable in part of the EGL (Figure 3g and 3h). After 34 weeks GSA EZH2 could not be detected in any layer of the human cerebellum (data not shown and Figure 3i).

**Figure 4:**
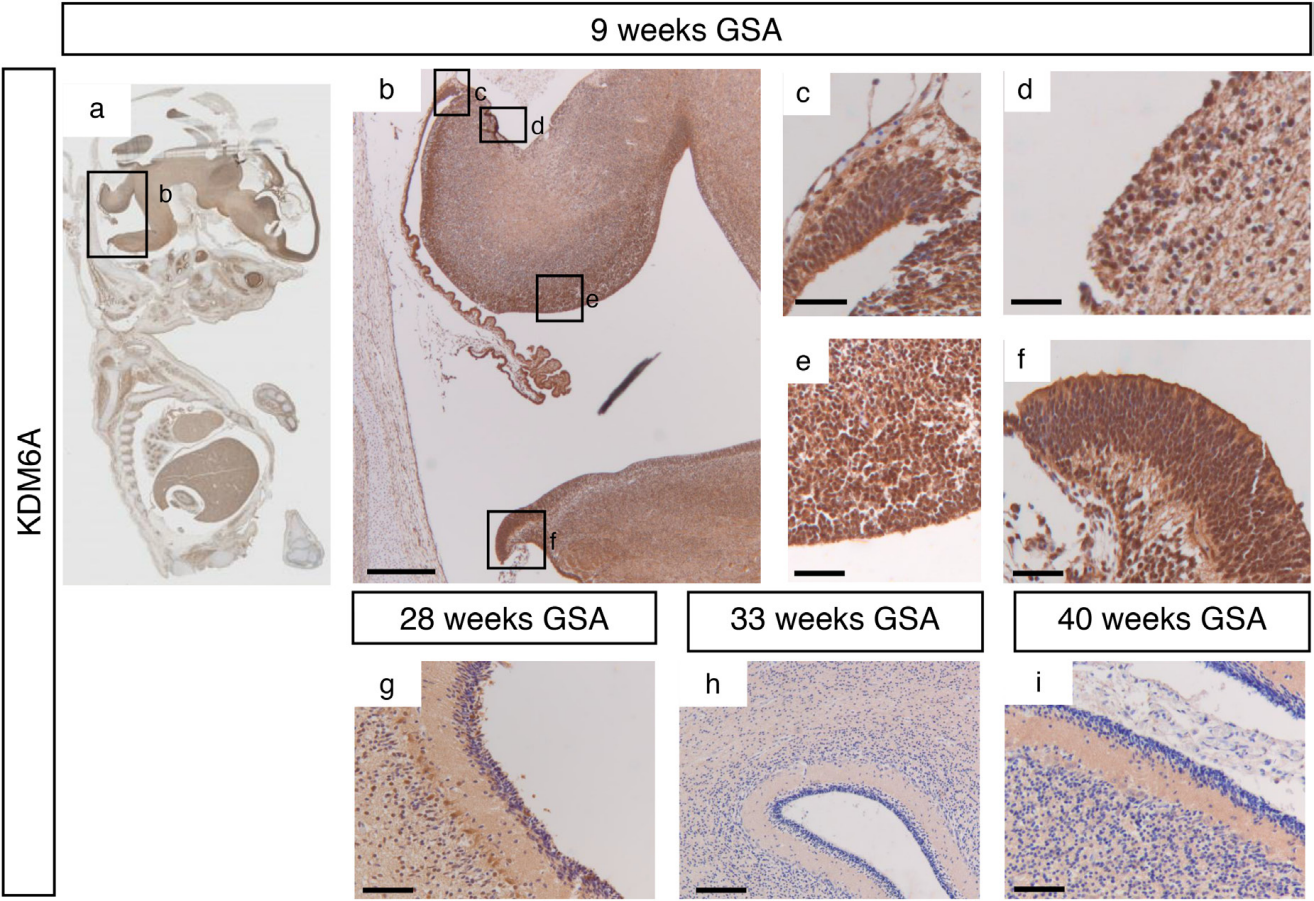
KDM6A expression during human cerebellum development **(a)** Expression of KDM6A expression at 9 weeks gestation. **(b-f)** Higher magnifications (400x) of boxed areas showing high KDM6A expression in the proliferative zones of the developing cerebellum; upper RL (c), EGL (d), VZ (e), and lower RL (f). At 28 weeks gestation, strong nuclear KDM6A expression was observed in Purkinje cells, and to a lesser extent in the granule neuron precursor cells In the EGL and in the granule cells in the IGL **(g)**. After 28 weeks GSA KDM6A could not be detected in any layer of the human cerebellum **(h-i)**. Scale bars 1000 μm (b), 100 μm (g), 50 μm (h), 25 μm (c-f and i).

**Figure 5:**
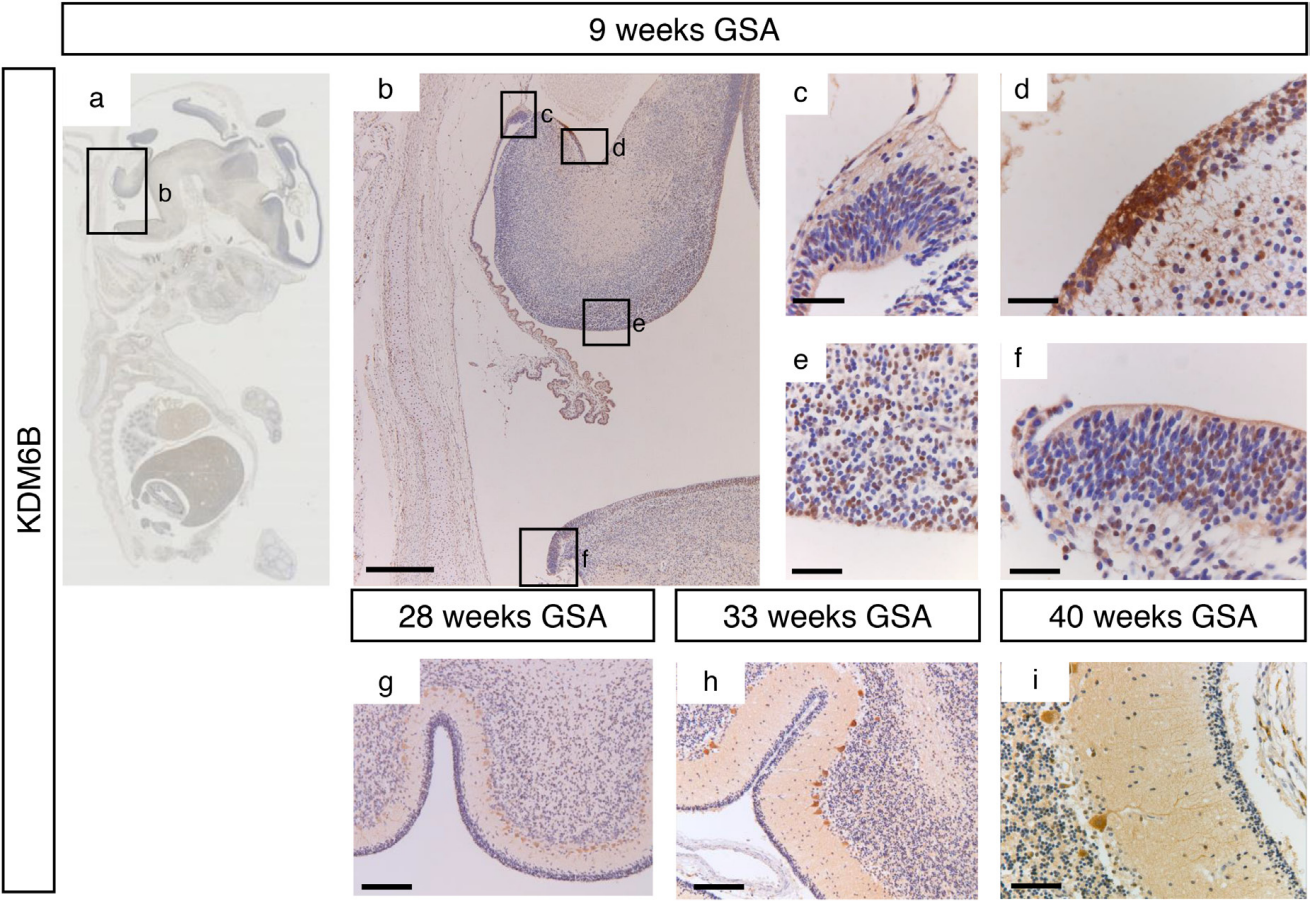
KDM6B expression during human cerebellum development **(a)** Expression of KDM6B at 9 weeks gestation. **(b-f)** Higher magnifications (400x) of boxed areas showing high KDM6B expression in the proliferative zones of the developing cerebellum; upper RL (c), EGL (d), VZ (e), and lower RL (f). During further development KDM6B is selectively expressed in the Purkinje cells. Staining intensity increases in time, and distribution changes from nuclear to more cytoplasmic (f-**i**). Scale bars 1000 μm (b), 100 μm **(g)**, 50 μm **(h)**, 25 μm (c-f and i).

During early cerebellar development (9 weeks GSA) an uniformly high protein expression for KDM6A was detected in all the proliferative zones (Figure 4a–4f and Supplementary Figure 1). At 28 weeks gestation, strong nuclear KDM6A expression was observed in Purkinje cells, and to a lesser extent in the granule neuron precursor cells In the EGL and in the granule cells in the IGL (Figure 4g). After 28 weeks GSA KDM6A could not be detected in any layer of the human cerebellum (Figure 4h–4i). KDM6B expression showed a bi-temporal expression pattern. During early cerebellar development (9 weeks GSA), KDM6B was present in the VZ, both upper and lower RL and EGL (Figure 5a–5f). However, compared to EZH2 and KDM6A fewer cells stained positive. Especially fewer KDM6B positive cells were present in both upper and lower RL. In contrast, KDM6B expression was more prominent in the VZ as compared to EZH2 (Figure 3e and 5e). Interestingly, the expression and localization of KDM6B changed during development. At 28 weeks gestation, expression of KDM6B was predominantly observed in Purkinje cells, and to a lesser extent in granule cells in the internal granule layer. Moreover, the staining intensity in Purkinje cells became more prominent in later stages and in adulthood (Figure 5g–5i and data not shown). The distribution of the signal also changed from only nuclear at 9 weeks GSA to predominantly cytoplasmic staining in Purkinje cells after 28 weeks GSA (Figure 5g–5i).

**Figure 6:**
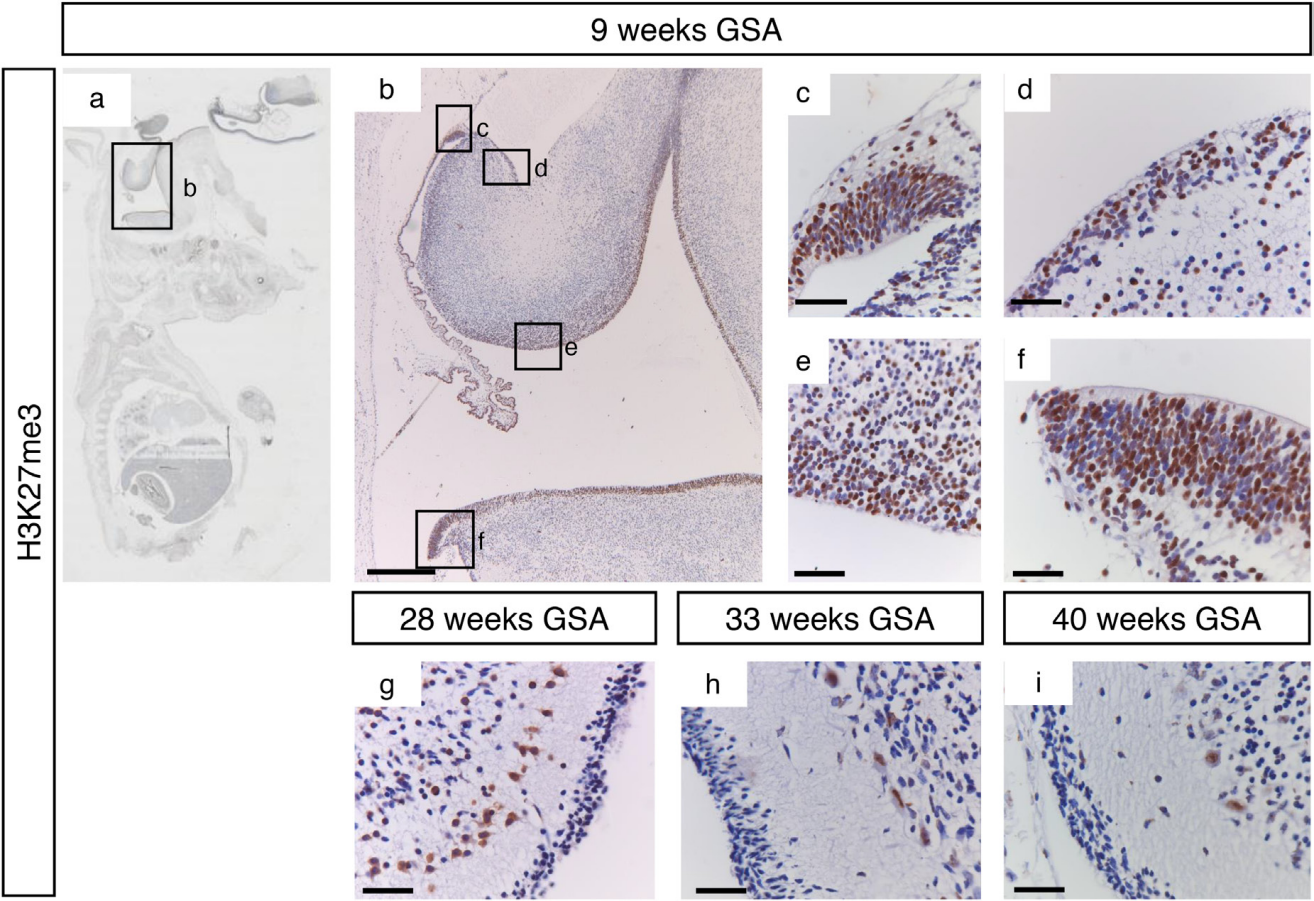
H3K27me3 trimethylation during human cerebellum development **(a)** Expression of H3K27me3 at 9 weeks gestation. **(b-f)** Higher magnifications (400x) of boxed areas showing high H3K27me3 expression in the proliferative zones of the developing cerebellum; upper RL (c), EGL (d), VZ (e), and lower RL (f). At 28 weeks gestation strong staining is present in Purkinje cells and some cells in the EGL and IGL layers **(g)**. From 33 weeks gestation and further H3K27me3 expression is only present in Purkinje cells **(h-i)**. Scale bars 1000 μm (B), 25 μm (c-i).

H3K27me3 showed a staining pattern that was comparable to EZH2 during early development (Figure 6a–6f). All proliferative zones showed strong H3K27me3 positive progenitor cells. During later development H3K27me3 staining was more localized in Purkinje cells, while a few positive cells could also be detected in the EGL and the internal granular layer (Figure 6g–6i). Whereas KDM6B staining in the Purkinje cells became more intense during development, H3K27me3 positive Purkinje cells became less intense (Figure 6g–6i). In contrary to the cytoplasmic localization of KDM6B in Purkinje cells, H3K27me3 staining was restricted to the cell nucleus (Figure 5i and 6i).

In conclusion, during development of the human cerebellum H3K27me3 and its regulators are expressed in a spatio-temporal manner. A summary of the expression patterns of EZH2, KDM6A and KDM6B and the trimethylation of H3K27 is given in Table 1.

**Table 1:** Summary of H3K27 regulators expression in the developing human cerebellum

	Week 9 GSA				Week 28 GSA				Week 33 GSA				Week 40 GSA			
	URL	EGL	VZ	LRL	EGL	ML	PCL	IGL	EGL	ML	PCL	IGL	EGL	ML	PCL	IGL
EZH2	+++	+++	+++	+++	+	-	-	+/-	+	-	-	-	-	-	-	-
KDM6A	+++	++	+++	+++	+	-	++	+/-	-	-	-	-	-	-	-	-
KDM6B	++	++	+++	++	-	-	++	+/-	-	-	+++	+/-	-	-	+++	+/-
H3K27me3	+++	+++	+++	+++	+	+/-	+	+	+	+/-	++	+	+/-	-	+/-	+

URL, upper rhombic lip; EGL, external granule layer; VZ, ventricular zone; LRL, lower rhombic lip; ML, molecular layer; PCL, Purkinje cell layer; IGL, internal granule layer. Relative staining intensity is indicated as: - absent staining; + / - weak and sporadic staining; + low intensity staining or strong sporadic staining; ++ medium intensity staining; +++ high intensity staining.

## DISCUSSION

Since the onset of MB is thought to be associated with a block in normal differentiation, and histone methylation patterns are frequently altered in MB, we investigated the pattern of H3K27me3 and its methylase EZH2 and demethylases KDM6A and KDM6B during human cerebellum development. Here, we provide evidence that during development of the human cerebellum H3K27me3 and its regulators are expressed in a spatio-temporal manner. Moreover, in line with previous studies, an aberrant expression pattern was seen in specific MB subgroups.

In recent years, four distinctive subgroups have been identified in MB: SHH, WNT, group 3, and group 4. Although an H3K27me3 enriched phenotype has been described exclusively for Group 3 and Group 4 MBs, amplification of EZH2 (gain of chromosome 7q) and deletion of KDM6B (loss of chromosome 17p) are also found in SHH MBs [23]. Moreover, both EZH2 and KDM6B have been linked to SHH signaling during cerebellar development [33]. The SHH subgroup is thought to arise from the EGL [8]. Here we observed temporal expression of H3K27me3, EZH2, KDM6A and KDM6B at 9 weeks gestation in the EGL. KDM6B became undetectable after 9 weeks GSA. Expression of EZH2, KDM6A, and H3K27me3 however decreased rapidly in the EGL layer, with only a few positive cells between 28 and 33 weeks gestation. Li *et al.*, have identified a subpopulation of Nestin positive progenitor cells (NEPs) within the EGL layer, which were found to be distinct from GNPs. When SHH signaling was aberrantly activated, these NEPs exhibited more severe genomic instability and gave rise to tumors more efficiently than GNPs [34]. EZH2 positive cells like the NEPs were present transiently during cerebellum development in the EGL (Figures 3f and g). It is tempting to speculate whether the EZH2 positive cells within the EGL cells between 28 and 33 weeks gestation could be in fact these NEPs.

For the WNT subgroup, progenitor cells of the lower RL have been identified as cells of origin, which normally give rise to the pontine grey matter [10]. During early human cerebellum development EZH2, KDM6A and KDM6B were highly expressed in the lower RL at 9 weeks gestation and high levels of H3K27me3 were detected. Several studies have addressed the important role of EZH2 and KDM6B in brainstem development [35, 36]. In mouse pre-cerebellar neurons, EZH2 expression controls proper pontine neuron migration [35]. Moreover, KDM6B controls appropriate organization of the Pre-Bötzinger complex, a cluster of interneurons in the brainstem which control the respiratory rhythm generator [36]. Results from both studies, showing high expression patterns for EZH2, KDM6B and H3K27me3 in progenitors of lower RL during mouse brain development, are in line with our results in the human cerebellum.

Group 3 and Group 4 MB more commonly show deregulation of H3K27 trimethylation as compared to the SHH or WNT MBs. These subgroups predominantly show somatic copy number aberrations and mutations of *EZH2*,*KDM6A* and *KDM6B* [23–25]. Seen from a developmental point of view, the GABAergic (Group 3) and glutamatergic (Group 4) gene profiles point toward possible different precursor cells of origin for these MBs. During development GABAergic neurons are derived from precursors of the VZ expressing *Pancreatic transcription factor 1a* (PTF1A) [1]. We have shown that H3K27me3, EZH2, KDM6A and KDM6B are temporally present in the VZ at 9 weeks gestation. Later during development, trimethylation of H3K27 and expression of KDM6A and KDM6B could be detected in the GABAergic derived Purkinje cells starting from 28 weeks GSA. Moreover, KDM6B expression became stronger during further development in the Purkinje cells. KDM6B expression in Purkinje cells shifted from nuclear to cytoplasmic, which might suggest that in Purkinje cells KDM6B is involved in demethylating non-histone proteins as previously shown for primary human fibroblasts [37]. Interestingly, the increase in KDM6B expression in Purkinje cells coincided with an increase in H3K27me3 in week 33, despite the absence of EZH2. Since it has been described previously that EZH2 deficient mouse models still show H3K27me2 and H3K27me3 marks [38, 39], this suggests that H3K27 methylation may occur through other methyltransferases, such as EZH1. According to the BrainSpan Atlas of the Developing Human Brain cerebellar transcript levels of EZH1 increase indeed at 25-38 weeks GSA (http://brainspan.org/).

Elevated levels of H3K27me3 in human cerebellum have been described in Ataxia-telangiectasia, a neurodegenerative disease. In the same study, H3K27me3 staining was shown to be low in Purkinje cells of healthy adults, in line with our results [40]. During development all glutamatergic neurons are derived from precursor cells of the upper RL [41, 42]. We detected strong expression of EZH2, KDM6A, KDM6B and H3K27me3 in the upper RL at 9 weeks gestation. Whether precursors of GABAergic and glutamatergic neurons drive Group 3 and Group 4 MB has recently received greater interest [13, 43]. The consistent high expression of EZH2 across different MB subgroups [30, 31, 44], and no expression of EZH2 after week 34 GSA, provides a possibility for targeted therapy with EZH2 inhibitors in medulloblastoma [31, 45, 46]. However, more preclinical experiments may be needed before implementing such inhibitors into clinical practice, as recent evidence suggests that deletion of EZH2 in group 3 tumors may also accelerate tumorigenesis [47].

In conclusion, our data show the spatio-temporal expression of H3K27me3 and its modifiers during human cerebellar development, and the comparison with the subtypes of MB indicate that the H3K27 methylation pathway is deregulated in MB.

## MATERIALS AND METHODS

### Fetal brain and MB tissue array samples

A database containing fetal autopsy cases that underwent a comprehensive neuropathological evaluation during the last 30 years was available at our hospital. We selected samples of cases between the gestational ages of 9–40 weeks and reviewed all Hematoxylin and Eosin stained sections and paraffin embedded blocks. Specimens were excluded if there were significant neuropathological findings, or if they showed any evidence of autolysis. At least two samples per gestational age were investigated. In addition, a largely independent tissue microarray (TMA) cohort with medulloblastoma specimen (n=87) was obtained from the files of the Department of Neuropathology of the Academic Medical Center (University of Amsterdam). Subgroup information was obtained by immunohistochemistry using antibodies for the subgroup-specific protein markers β-catenin (WNT), DKK1 (WNT), SFRP1 (SHH), NPR3 (Group 3), and KCNA1 (Group 4) as described by Northcott *et al.* [48]. Informed consent was obtained according to institutionally-approved protocols.

### Immunohistochemistry

Immunohistochemical staining was performed on formalin-fixed, paraffin-embedded 4-μm sections of tissue of the various developmental stages. For all sections, heat induced antigen retrieval was carried out in a microwave in a 10 mM citrate buffer (pH 6). Endogenous peroxidase was blocked for half an hour in 0.3% H_2_O_2_ in methanol. Afterwards, sections were stained for monoclonal mouse anti-EZH2 (1:200; cat. NCL-L-EZH2, Novacastra), polyclonal rabbit anti-KDM6A (1:750; cat. HPA001165, Sigma Aldrich), polyclonal mouse anti-KDM6B, C-terminus (1:200; cat. AP1022b, Abgent), and monoclonal rabbit anti-H3K27me3 (1:200; cat. 9733, Cell Signaling), all diluted in standard antibody diluents. As a negative control, the staining protocol was followed without adding primary antibodies. As a positive control tonsil tissue was stained for EZH2 and H3K27me3, placental tissue for KDM6A and neocortex tissue for KDM6B. Primary antibodies EZH2 were incubated for 30 min. Primary antibodies KDM6A, KDM6B and H3K27me3 were incubated overnight. DAB was used as a substrate for the peroxidase based Envision detection system (cat.K4065, DAKO, Heverlee, Belgium). Slides were counterstained with Hematoxylin.

### Datamining

Immunohistochemical stainings were compared to mRNA expression of *EZH2*, *KDM6A*, and *KDM6B* during human embryonal development described by Miller *et al.* [32], using the R2 genomics analysis and visualization platform (http://r2.amc.nl): normal brain development – brain span – 524 – rpkm – brspv10rs. Time points used are stage 2A (early prenatal, corresponding with 9 weeks GSA), stage 5 (late prenatal, corresponding with 28-33 weeks GSA), and stage 6 (birth-early infancy, corresponding with 40 weeks GSA).

## SUPPLEMENTARY MATERIALS FIGURE



## Abbreviations

MB: medulloblastoma
EZH2: Enhancer of zeste homolog 2
H3K27(me3): histone 3 lysine 27 (trimethylation)
KDM6A: Lysine-specific demethylase 6A
KDM6B: Lysine-specific demethylase 6B
WNT: Wingless-type MMTV integration site family, member 1
SHH: Sonic Hedgehog
VZ: ventricular zone
RL: rhombic lip
GNPs: granule precursor cells
EGL: external granule layer
IGL: internal granule layer
ML: molecular layer
PCL: Purkinje cell layer
NEPs: nestin positive precursor cells
GSA: gestational age
