# Supplementary material for: Trimethylation of H3K27 during human cerebellar development in relation to medulloblastoma
Source: Oncotarget. 2017 Sep 8;8(45):78978–88. doi: 10.18632/oncotarget.20741 (PMC5668013; doi:10.18632/oncotarget.20741)
Supplement: Supplementary file 1 [file oncotarget-08-78978-s001.pdf]

Trimethylation of H3K27 during human cerebellar development in relation to medulloblastoma

SUPPLEMENTARY MATERIAL

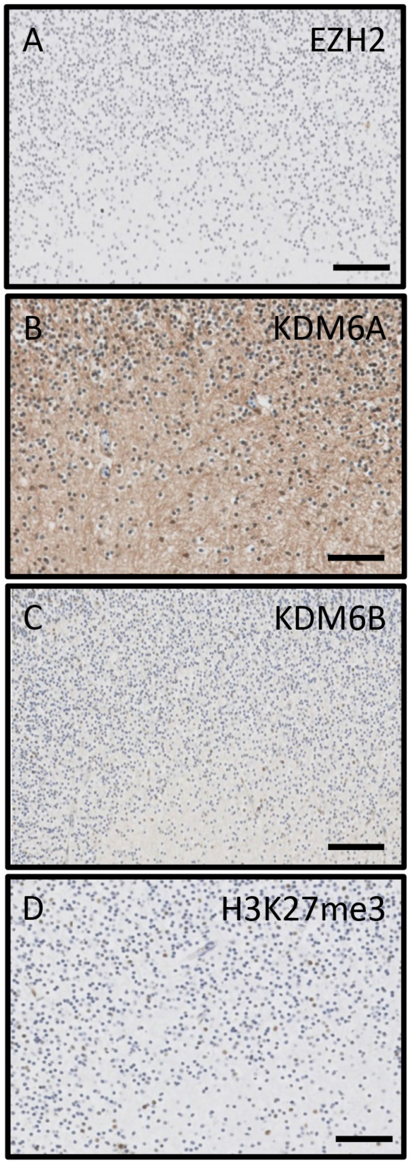

Supplymentary Figure 1: Expression of H3K27me3 and its modifiers at 9 weeks GSA in the ventricular zone. Shown are expression of EZH2 **(A)**, KDM6A **(B)**, KDM6B **(C)**, and H3K27me3 **(D)** in the ventricular zone, comprising mostly negatively stained granular cells. Scale bar size=25µm.
